# Contrasting Effects of Phosphate on Arsenic Mobility and Human Exposure in Agricultural Soils

**DOI:** 10.1029/2026GH002031

**Published:** 2026-09-15

**Authors:** Petr Drahota, Alena Černíková, Vojtěch Ettler, Johana Kešnerová, Martin Lichovník, Martin Mihaljevič

**Affiliations:** ^1^ Institute of Geochemistry, Mineralogy and Mineral Resources Faculty of Science Charles University Prague Czech Republic; ^2^ Institute of Applied Mathematics and Information Technologies Faculty of Science, Charles University Prague Czech Republic; ^3^ Department of Mathematics Faculty of Science Jan Evangelista Purkyně University in Ústí nad Labem Ústí nad Labem Czech Republic

**Keywords:** arsenic, bioaccessibility, agricultural soils, phosphate, Fe (oxyhydr)oxides, dust exposure

## Abstract

Arsenic in agricultural soils may pose human health risks due to elevated concentrations and increased soil ingestion associated with dust generation. However, the actual exposure depends on the fraction of As that becomes bioaccessible under gastrointestinal conditions. This study evaluates As mobility, bioaccessibility, and associated health risks in 57 agricultural topsoils from seven regions affected by geogenic enrichment and historical mining. Arsenic partitioning was characterized using operationally defined extractions and mineralogical analyses, and bioaccessibility was determined by an in vitro gastric assay. The total As concentrations range from 25 to 2007 mg kg^−1^, exceeding national agricultural soil thresholds (40 mg kg^−1^) in ∼80% of samples. Nevertheless, relative As bioaccessibility ranges from 2% to 38%, with most soils exhibiting values below 10%. Arsenic is predominantly associated with Fe (oxyhydr)oxides, while discrete As‐bearing minerals are rare. Surface‐bound As, defined by phosphate extraction, strongly controls both mobility and bioaccessibility. Long‐term phosphate enrichment due to repeated fertilization exerts contrasting effects on As behavior. Readily soluble P enhances both As mobility and bioaccessibility, whereas higher Olsen‐extractable P is associated with lower As bioaccessibility, suggesting progressive occupation of reactive sorption sites. A risk assessment based on a conservative rural ingestion scenario indicates that most soils did not exceed non‐carcinogenic thresholds when bioaccessible As was considered. These findings demonstrate that bioaccessible As and associated exposure risk are controlled primarily by reactive surface‐bound As fractions rather than by total soil As concentrations, whereas phosphate exerts contrasting secondary effects depending on its geochemical availability and partitioning.

## Plain Language Summary

Arsenic is a toxic element that occurs naturally in some soils and can also accumulate because of historical mining and smelting. In agricultural areas, people may be exposed to arsenic by accidentally ingesting soil particles during agricultural activities or by inhaling dust generated during tillage and wind erosion. However, the health risk depends not only on the total amount of arsenic in soil but also on how much of it can dissolve in the human digestive system. We studied agricultural soils from seven regions in the Czech Republic affected by natural arsenic enrichment and historical mining. We measured arsenic concentrations in different soil particle sizes, evaluated how much arsenic becomes available under simulated stomach conditions, and examined the role of phosphate derived from long‐term fertilization. Although many soils contained high arsenic concentrations, only a relatively small fraction was bioaccessible. Arsenic bioaccessibility was controlled mainly by reactive arsenic bound to iron minerals. Phosphate showed contrasting effects: readily available phosphate promoted arsenic release, whereas long‐term phosphate accumulation was associated with lower arsenic bioaccessibility. These findings improve exposure assessment and environmental health evaluation in agricultural areas affected by arsenic contamination.

## Key Points

- Surface‐bound arsenic controls bioaccessibility more strongly than total soil arsenic
- Phosphate exerts contrasting effects on arsenic mobility and human exposure
- Most agricultural soils showed low exposure risk despite elevated arsenic levels

## 1 Introduction

Arsenic (As) is a potentially carcinogenic metalloid ubiquitously distributed in various forms in the soil environment. When present in contaminated soil, the incidental oral ingestion of soil particles is a primary exposure pathway for As (Calabrese et al., 1997; LaGoy, 1987), and the actual health risk depends on the magnitude, frequency, and duration of exposure. The magnitude of exposure is influenced not only by the concentration of As in the soil matrix but also by its bioaccessibility (i.e., the fraction of As solubilized in gastrointestinal fluids and available for absorption into the systemic circulation). Another critical factor influencing human exposure is the soil ingestion rate, which varies with the lifestyle, environmental conditions, and land use. Agricultural activities such as plowing, tilling, and harvesting can increase exposure to airborne and resuspended soil particles, thereby enhancing incidental soil ingestion and dust inhalation in rural environments (Doyle et al., 2010; Hubbard et al., 2022; Jia et al., 2023; Nieuwenhuijsen et al., 1998; Shenker, 2000). These activities also reduce soil resistance to wind erosion, increasing fugitive dust emissions and the dispersal of contaminants (Kang et al., 2021; Smith & Lee, 2003). Agricultural populations may represent a distinct exposure group because repeated contact with cultivated soils, dust generation, and residential proximity to agricultural land can increase incidental ingestion and inhalation pathways. Despite this, agricultural exposure scenarios remain underrepresented in soil health risk studies.

Arsenic is one of the most common metal(loid) contaminants found in agricultural soils (Hou et al., 2020). The highest concentrations are typically observed in regions associated with sulfide mineralization (Kwon et al., 2012; Tarvainen et al., 2013; Zhou et al., 2018), where subsequent mining and smelting activities have frequently intensified and spatially expanded the contamination within these geogenic anomalies. In addition to other common anthropogenic sources of As in the pedosphere, such as industrial emissions and coal combustion, the largest sources of As in agricultural soils are commercial agricultural products, including pesticides/insecticides, herbicides, and phosphate fertilizers (Smith et al., 1998). While As in these soils may be partially incorporated into the residues of arsenical agricultural chemicals (Cancès et al., 2008), the majority is bound to a variety of soil minerals and substrates. In most soils, As is predominantly associated with metal (oxyhydr)oxides, showing the highest affinity for Fe(III) (oxyhydr)oxides (Dixit & Hering, 2003; Stollenwerk, 2003; Tiberg et al., 2020). These phases are often incorporated into small soil aggregates composed of clay minerals and organic matter, which may also play a role in the sorption and transport of As in the soil environment (Goldberg, 2002; Wang & Mulligan, 2006).

The in vitro bioaccessibility (IVBA) of As in soils and dust varies widely and is strongly influenced by solid‐phase speciation and geochemical partitioning (Basta & Juhasz, 2014; Bradham et al., 2018; Sowers et al., 2022). Arsenic associated with Fe (oxyhydr)oxides generally exhibits low solubility (≤5%) under the gastrointestinal conditions (Beak et al., 2006; Ehlert al., 2018) and can be retained or re‐adsorbed during IVBA extraction (Sowers et al., 2022). Consequently, numerous studies have linked lower As bioaccessibility to higher total Fe contents and stronger association with Fe (oxyhydr)oxide phases (Juhasz et al., 2007; Mikutta et al., 2014; Palumbo‐Roe et al., 2015; Smith et al., 2008; Sowers et al., 2022; Yang et al., 2002). The efficiency of As sorption in IVBA assays depends on multiple factors such as pH, As speciation, ionic strength, and competing ions, with phosphate being the main competitor for the sorption sites (Bradham et al., 2018; Sowers et al., 2022). The addition of phosphate to IVBA assays has been shown to enhance As bioaccessibility through the competitive desorption of As from Fe (oxyhydr)oxide surfaces (Basta et al., 2007; Brattin et al., 2013; Li et al., 2017). However, these studies are based on short‐term phosphate amendments conducted under controlled laboratory conditions and often at relatively high phosphate concentrations. In contrast, agricultural soils are typically exposed to long‐term and repeated phosphate inputs through fertilization, which may progressively modify the partitioning of As, sorption equilibria, and the reactivity of mineral surfaces over decadal timescales. Despite previous experimental evidence for phosphate‐induced As mobilization, field‐based studies linking long‐term agricultural phosphate accumulation with As mobility, bioaccessibility, and exposure risk remain limited.

Together, these findings suggest that agricultural activities on contaminated land can influence the human exposure to As through both elevated soil ingestion rates and phosphate‐influenced bioaccessibility. Nevertheless, the systematic evaluation of these processes in historically contaminated agricultural soils under realistic field conditions remains limited. To address this knowledge gap, we investigated the effect of naturally accumulated phosphate on the As mobility and bioaccessibility in arable soils located near residential sites and characterized by contrasting contamination sources and soil properties. We hypothesize that the long‐term agricultural phosphate enrichment modifies the partitioning of As between relatively stable and more reactive surface‐associated pools, thereby influencing As mobility and human exposure potential. Specifically, we combined in vitro bioaccessibility assays with physicochemical and mineralogical analyses to (a) characterize the As partitioning across exposure‐relevant particle size fractions, (b) identify the dominant geochemical controls influencing As mobility and bioaccessibility, with particular emphasis on phosphate pools characterized by selective chemical extractions, and (c) evaluate the implications of As–P interactions for exposure assessments in agricultural soils.

## 2 Materials and Methods

### 2.1 Soil Sampling and Characterization

Seven agricultural regions in the central Czech Republic, known for elevated concentrations of As in soils, were selected for this study: Kutná Hora, Příbram, Havlíčkův Brod, Smolotely‐Líšnice, Mokrsko, Příčovy–Krásná Hora and Tehov (Figure S1 in Supporting Information S1). The elevated As levels in these regions result from the natural weathering of As‐rich bedrock (Mokrsko and Tehov) or from a combination of natural weathering and historical mining and smelting activities. The regions encompass diverse geological settings with variable bedrocks, including granodiorites, gneisses, and phyllites, often containing hydrothermal ore deposits of Ag, Pb, Zn, Cu, Sb, W, or Au with mining histories ranging from the medieval period to modern times (see Drahota et al., 2026 for detailed site descriptions). The climate is temperate, with a moderate annual rainfall (500–700 mm) and mean annual temperatures of 8–9°C.

The sampling locations were spatially distributed across the arable land within each region to ensure representative regional coverage and to capture the variability in the parent material and the intensity of contamination. A total of 57 composite topsoil samples were collected from the well‐aerated plowing layer (0–20 cm), representing the actively cultivated horizon relevant for agricultural exposure. Each composite sample consisted of nine subsamples taken from a 400 m^2^ area, with 6–14 samples collected per region to capture the spatial heterogeneity. The soils were air‐dried and sieved to <2,000 μm, <150 μm (PM_150_), and <10 μm (PM_10_), representing bulk soil, exposure‐relevant hand‐to‐mouth particles, and inhalable dust fractions, respectively. Sieving was performed using a vibratory sieve shaker (AS 200, Retsch) and a polyamide sieve with a nominal mesh size of 10 μm (SEFAR NITEX 03–10/2, SEFAR AG). Representative portions of fractions <2,000 μm and PM_150_ were finely ground using a planetary ball mill (PM400, Retsch).

The soil pH was measured in deionized water and 1 M KCl solutions (soil‐to‐solution ratio = 1:2.5) using a SenTix41 electrode and a WTW multimeter. The total sulfur, organic carbon, and inorganic carbon were determined with Eltra CS 500 and CS 530 TIC analyzers (ELTRA). The total element content (Al, As, Ca, Fe, K, Mg, Mn, Na, P) in each particle size fraction was analyzed by inductively coupled plasma optical emission spectrometry (ICP‐OES; Agilent 5100) after microwave digestion (Multiwave 5000, Anton Paar) with concentrated HNO_3_ and HF (3:1). The accuracy of the digestion procedure was assessed using the National Institute of Standards and Technology (NIST) standard reference materials 2710a and 2711a (Montana soils; Table S1 in Supporting Information S2). The precision of the duplicate digestions of both samples and reference materials was better than 9% for most elements (except for Al and Mg, which were within 21%).

The potentially mobile As and P fractions, together with the surface‐bound As in the bulk soils (<2,000 μm), were extracted in duplicate with 0.05 M (NH_4_)_2_SO_4_ and 0.05 M (NH_4_)_2_HPO_4_ solutions (soil‐to‐solution ratio = 1:25) for 4 and 16 hr, respectively (Wenzel et al., 2001). The readily exchangeable P pool in the bulk soil was determined by Olsen extraction with 0.5 M NaHCO_3_ for 30 min (Sims, 2000). The Olsen extraction was used to estimate relatively labile and agronomically relevant phosphate pools associated with long‐term fertilization. The As and P bound to the poorly crystalline metal (oxyhydr)oxides in each of the three particle‐size fractions were extracted with 0.2 M NH_4_‐oxalate (2 hr in the dark; soil‐to‐solution ratio = 1:100, pH 3) (Schwertmann, 1964). All the extracts were analyzed by ICP‐OES after 0.2‐μm membrane filtration (Nylon). The precision of the duplicate extractions was better than 12%.

The bulk mineralogy of the fraction <2,000 μm and PM_10_ was characterized by X‐ray diffraction (XRD) using a PANalytical X’Pert Pro diffractometer. Detailed analytical conditions and results have been previously reported (Drahota et al., 2026). Representative PM_150_ samples from each region were also prepared as polished sections and analyzed using a field emission electron probe microanalyzer (FEG‐EPMA, JEOL JXA‐8530F). The analytical conditions, reference standards, and detection limits are provided in Table S2 in Supporting Information S2.

### 2.2 Bioaccessibility of Arsenic

Major exposure pathways that contribute to incidental soil ingestion in rural agricultural environments include hand‐to‐mouth transfer of soil particles during agricultural activities and inhalation of fine fugitive dust generated by wind erosion or tilling operations (Smith & Lee, 2003). According to the United States Environmental Protection Agency (US EPA), PM_150_ particles are most likely to adhere to the hands and subsequently be ingested (US EPA, 2017), whereas inhaled PM_10_ particles are largely cleared from the respiratory tract and transported to the gastrointestinal tract via the mucociliary escalator (Carvalho et al., 2011). The combined evaluation of particle‐size distribution and bioaccessible As was therefore intended to better approximate realistic oral exposure from agricultural soils.

The in vitro bioaccessible As in PM_10_ and PM_150_ was determined using an in vitro method developed by the Solubility/Bioavailability Research Consortium assay (Kelley et al., 2002), which was later adopted by the US EPA (US EPA, 2017). This assay is widely applied in human health risk assessment and has been extensively validated against in vivo relative bioavailability measurements (Bradham et al., 2015; Juhasz, Herde, et al., 2014). The As IVBA data from this assay show a strong correlation with the As relative bioavailability determined in animal models, including mice and swine (Juhasz et al., 2009; Juhasz, Smith, et al., 2014). Briefly, the samples were added to 0.4 M glycine adjusted to pH 1.5 with concentrated HCl, at a soil‐to‐solution ratio of 1:100. The suspensions were gently shaken for 1 hr at 37°C in a GFL 3032 incubator. Subsequently, the suspensions were filtered through 0.45 μm nylon filters and the filtrates were analyzed using ICP‐OES and inductively coupled plasma mass spectrometry (ICP‐MS, Agilent 8900, USA) with a collision reaction cell. The bioaccessibility of As and the extractability of P and Fe were expressed both as absolute concentrations (As and P in mg kg^−1^, Fe in g kg^−1^) and as percentages of their total content in the corresponding particle‐size fraction. The extracts were performed in duplicate for each test soil and fraction. Quality control (QC) standards, including reagent blanks, blank spikes, and reference materials (NIST SRM 2710a and 2711a), were included with each batch. All the QC sample results were within acceptable limits as defined by Dodd et al. (2024).

### 2.3 Exposure and Risk Assessment

Chronic exposure to As through incidental soil ingestion was evaluated for adult residents living in rural areas with ongoing agricultural activities, representing a realistic high‐frequency exposure scenario. The assessment followed the standard US EPA methodology for estimating the chronic daily intake (CDI), cancer risk (CR), and hazard quotient (HQ) for oral exposure (US EPA, 1989). The exposure parameters were selected to represent a conservative rural scenario consistent with the regulatory risk assessment practice: soil ingestion rate of 100 mg day^−1^, exposure frequency of 250 days year^−1^, exposure duration (ED) of 25 years, average adult body weight of 70 kg, and average time of 25 550 days (70 years) for carcinogenic effects and ED × 365 days for non‐carcinogenic effects. The oral cancer slope factor (SF) for As was set at 1.5 (mg kg^−1^ day^−1^), and the oral reference dose (RfD) at 3 × 10^−4^ mg kg^−1^ day^−1^ (US EPA, 2007). The cancer risk was obtained by multiplying the CDI by the SF, while the HQ was calculated as the ratio of CDI to RfD.

### 2.4 Data Processing

Data were processed and visualized using DataGraph 5 (Visual Data Tools, USA). All the statistical analyses were performed in R 4.5.1 (RStudio interface). The data distribution was assessed using descriptive statistics, Q–Q plots, and the Shapiro–Wilk test. Because most variables did not meet the normality assumptions, non‐parametric tests (Wilcoxon test, Kruskal–Wallis test followed by Dunn's post‐hoc comparisons) were applied. The relationships between the variables were evaluated using Spearman's rank correlation. In addition, linear regression models, including models with locality and log‐transformed variables, were fitted as complementary tools, and multiple linear regression models were refined using stepwise selection based on Akaike's Information Criterion. The statistical significance was set at *α* = 0.05. Stepwise model selection was used as an exploratory tool, and regression results were interpreted primarily in conjunction with the non‐parametric correlations, geochemical plausibility, and consistency across complementary statistical approaches.

## 3 Results

### 3.1 Soil Characterization

The soils were classified mainly as Cambisols (Table S3 in Supporting Information S2). The proportion of PM_150_ ranged from 13% to 50% (mean *x̅* = 28%), while PM_10_ ranged from 1% to 13% (*x̅* = 5.1%) (Figure S2a in Supporting Information S1). Higher proportions of fine particles were observed in the soils developed on gneiss (Kutná Hora, Příbram, Havlíčkův Brod and Tehov), relative to the soils derived from granodiorite (Smolotely–Líšnice and Mokrsko). The soil pH ranged from 4.6 to 7.6 (*x̅* ± *σ*: 5.87 ± 0.74), with the highest values recorded at Kutná Hora (*x̅* ± *σ*: 7.15 ± 0.39) and the lowest at Havlíčkův Brod (*x̅* ± *σ*: 5.13 ± 0.34) (Figure S2b in Supporting Information S1). The elevated pH at the Kutná Hora region was associated with inorganic carbon (≤2.4 g kg^−1^), which is consistent with the presence of calcite identified by X‐ray diffraction (XRD) (Drahota et al., 2026). The organic carbon contents varied between 10 and 32 g kg^−1^, with the comparable median values among the regions (12–17 g kg^−1^).

XRD analyses showed that the bulk mineralogy was dominated by quartz, feldspars, and phyllosilicates, while PM_10_ was consistently enriched in clay minerals relative to coarser fractions (Drahota et al., 2026). Crystalline As‐ or P‐bearing minerals were not detected by XRD, except for minor minerals of the jarosite‐group identified in two samples affected by dispersed mine waste (KH3 and HB23; Table S3 in Supporting Information S2), which may incorporate As and P in their structure (Hudson‐Edwards, 2019).

### 3.2 Arsenic and Phosphorus in Bulk Soils and Size Fractions

**Figure 1:**
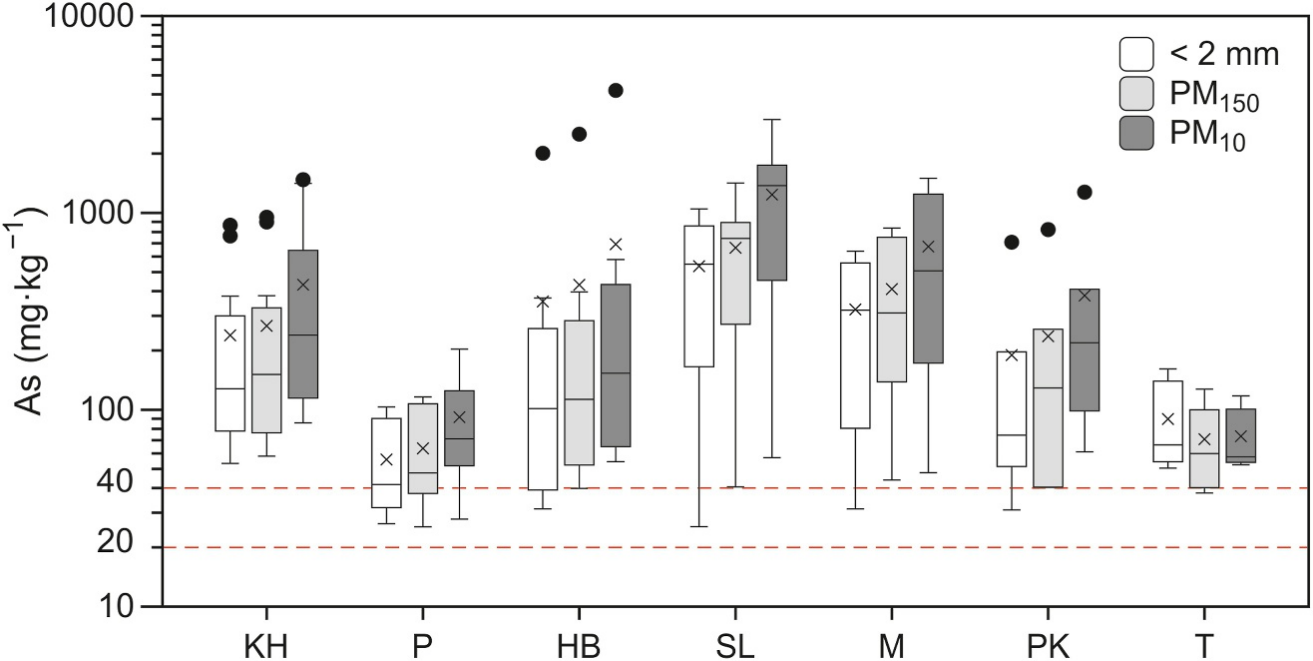
Box‐and‐whisker plot showing the variability of the As concentrations in 57 soil samples in three different particle size fractions (<2 mm, PM_150_ and PM_10_). The study regions include Kutná Hora (KH), Příbram (P), Havlíčkův Brod (HB), Smolotely‐Líšnice (SL), Mokrsko (M), Příčovy (PK), and Tehov (T). The colored horizontal lines indicate the preventive limit (20 mg kg^−1^) and the indicative limit (40 mg kg^−1^) for As in agricultural soils in the Czech Republic (MECR, 2016). The box plots show the median (horizontal line), interquartile range (IQR; box), minimum and maximum values within 1.5× IQR (whiskers), mean (cross) and outliers (individual points).

The total concentration of As in the soils (*n* = 57) ranged from 25 to 2007 mg kg^−1^ (*x̅* = 274 mg kg^−1^; Table S3 in Supporting Information S2), with the majority of samples exceeding the national preventive and indicative thresholds for agricultural soils (Figure 1). The As concentrations generally increased with decreasing particle size, thereby enriching the exposure‐relevant PM_150_ and PM_10_ fractions (Figure 1; Table S3 in Supporting Information S2). The operationally defined extractions of bulk soils showed that the sulfate‐extractable As (As_SO4_), representing a potentially mobile fraction, accounted for 0.1%–4.4% of the total As, whereas the phosphate‐extractable As (As_PO4_), interpreted as a surface‐bound fraction, represented 1.8%–23.7% (Figure S3a in Supporting Information S1; full numerical results in Table S3 in Supporting Information S2). In most regions, these fractions were relatively low and spatially consistent. In contrast, the soils from Kutná Hora exhibited distinctly elevated proportions of both sulfate‐extractable and surface‐bound As (*x̅* ± *σ*: As_SO4_ = 1.7 ± 1.2%; As_PO4_ = 18.7 ± 3.6%), which strongly contributed to the overall variability (CV ≥ 87% for all the data).

The total concentration of P in the bulk soils ranged from 500 to 2,950 mg kg^−1^ (*x̅* = 953 mg kg^−1^). In 90% of the samples, P was enriched in PM_150_ and PM_10_ relative to the bulk soil, without a systematic regional trend (Figure S4 in Supporting Information S1). The readily soluble P (P_SO4_) accounted for 0.4%–3.3% of the total P, whereas the Olsen‐extractable P (P_OLSEN_) ranged from 2.7% to 12.6% (Figure S3b in Supporting Information S1). Unlike the elevated As mobility in Kutná Hora, the P mobility showed little variation across the regions (CV ≤ 54% for all the data).

### 3.3 Solid‐Phase Arsenic and Phosphorus

The oxalate extraction indicated that a substantial proportion of both As and P is associated with poorly crystalline Fe (oxyhydr)oxides and other reactive phases, ranging from 10% to 83% for As (*x̅* = 36%) and from 22% to 71% for P (*x̅* = 43%) (Figure S5 in Supporting Information S1). The variability in the oxalate‐extractable As (CV = 59%) was markedly higher than that of P (CV = 26%), primarily due to the elevated As association with these phases in Kutná Hora (*x̅* ± *σ*: 62 ± 12%), whereas other regions showed considerably lower proportions (*x̅* ± *σ*: 23 ± 10%). The proportion of both elements in the oxalate‐extractable fractions increased with the decreasing particle size, reflecting the enrichment of reactive Fe (oxyhydr)oxides in the finer fractions (Figure S5 in Supporting Information S1). This is supported by similar molar Fe/As and Fe/P ratios in the oxalate extraction across different particle‐size fractions (Figure S6 in Supporting Information S1).

**Figure 2:**
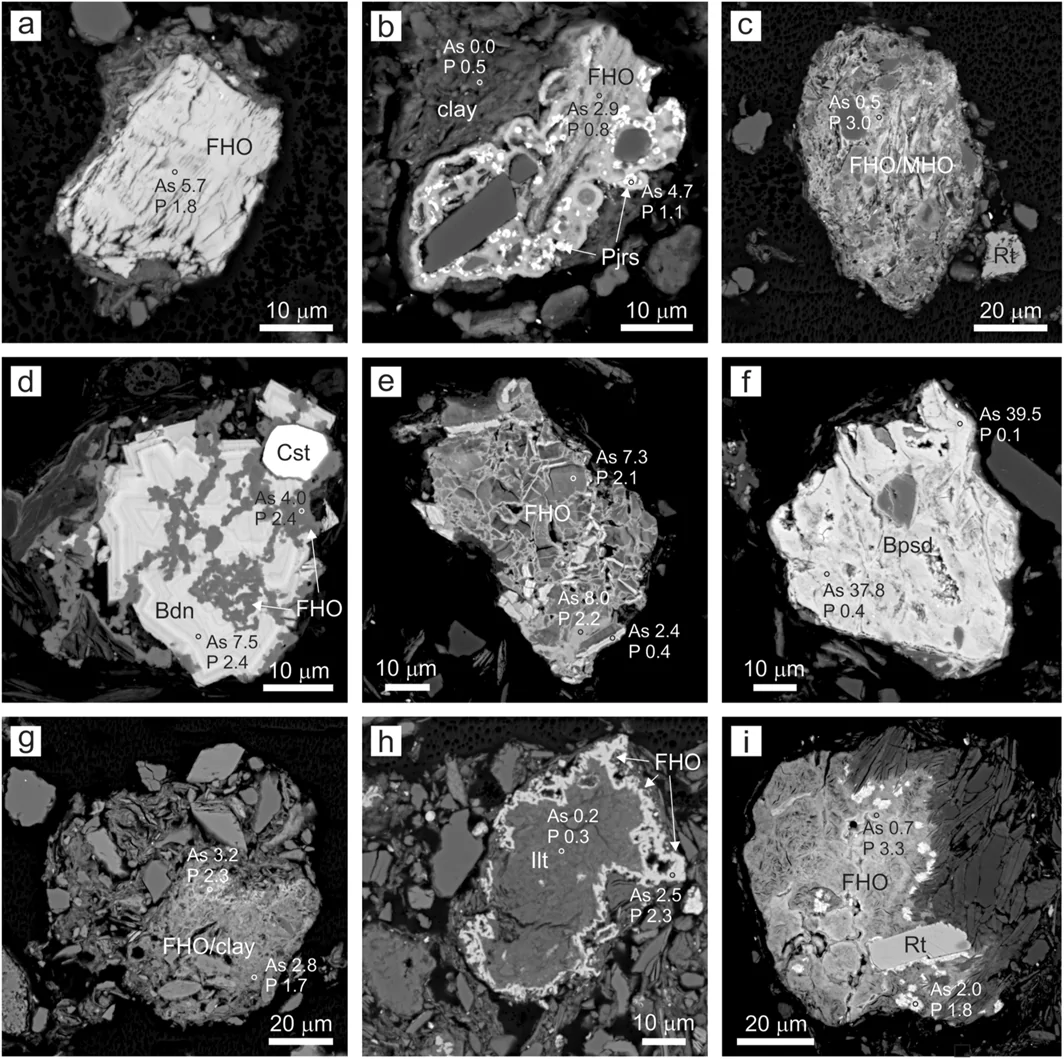
Back‐scattered electron SEM images of the As‐bearing phases from agricultural soils collected at Kutná Hora (a, b), Příbram (c), Havlíčkův Brod (d), Smolotely‐Líšnice (e, f), Mokrsko (g), Příčovy (h) and Tehov (i). The As and P concentrations are expressed in wt% as oxides (As_2_O_5_ and P_2_O_5_). Bdn—beudantite, Bpsd—bariopharmacosiderite, Cst—cassiterite, FHO—Fe (oxyhydr)oxide, Ilt—illite, MHO—Mn (oxyhydr)oxide, Pjrs—plumbojarosite, Rt—rutile.

A detailed EPMA investigation of the representative PM_150_ samples confirmed that Fe (oxyhydr)oxides are the dominant carriers of As and P (Figure S7 in Supporting Information S1), accounting for more than 80% of the As‐bearing grains (94 grains examined). These phases occur predominantly within the heterogeneous soil aggregates composed of aluminosilicates, organic matter and Mn (oxyhydr)oxides, and less frequently as discrete grains (Figure 2). The As concentrations in Fe (oxyhydr)oxides were highly variable (≤17.3 wt% As_2_O_5_), whereas the P concentrations were more consistent (≤6.4 wt% P_2_O_5_). In contrast, clay minerals contained only minor As and P (≤0.5 wt% As_2_O_5_ and P_2_O_5_) (Figures 2b–2h). Other As‐bearing minerals were detected only locally and in minor amounts, including bariopharmacosiderite [Ba_0.5_Fe_4_(AsO_4_)_3_(OH)_4_·5H_2_O] of geogenic origin (Figure 2f) and Pb‐bearing hydroxysulfates and arsenates from the alunite supergroup (Figures 2b–2d) in the soils affected by mining activities. Apatite [Ca_5_(PO_4_)_3_(Cl, F, OH)] represented a minor P‐bearing mineral in all the samples.

### 3.4 Bioaccessibility of Arsenic and Extractability of Iron and Phosphorus

**Figure 3:**
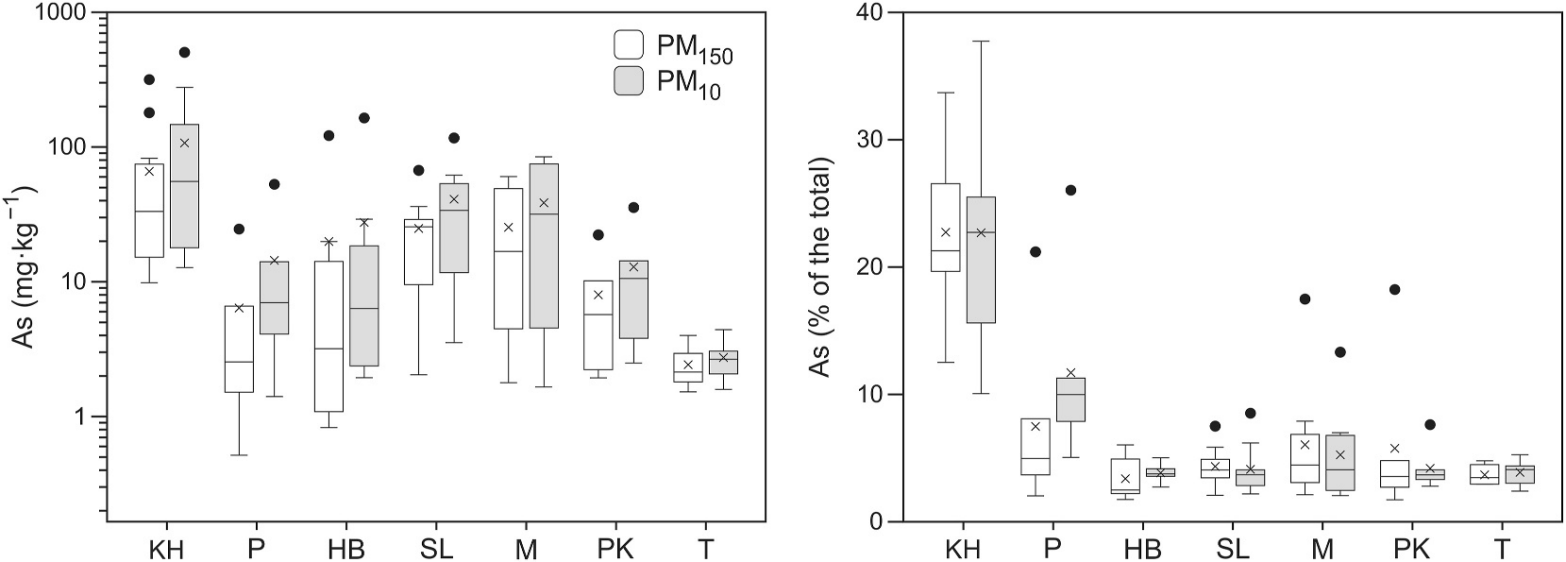
Box‐and‐whisker plots showing the variability in the total and relative As bioaccessibility across size fractions (PM_150_ and PM_10_) of 57 soil samples from Kutná Hora (KH), Příbram (P), Havlíčkův Brod (HB), Smolotely‐Líšnice (SL), Mokrsko (M), Příčovy (PK), and Tehov (T). The plots display the median (horizontal line), interquartile range (box), minimum and maximum values within 1.5× IQR (whiskers), mean (cross) and outliers (individual points).

The absolute As IVBA ranged from 0.5 to 316 mg kg^−1^ (*x̅* = 28 mg kg^−1^) in PM_150_ and from 1.4 to 504 mg kg^−1^ (*x̅* = 45 mg kg^−1^) in PM_10_, corresponding to 1.7%–34% (*x̅* = 9.4%) and 2.1%–38% (*x̅* = 9.6%) of the total As, respectively (Figure 3, Table S3 in Supporting Information S2). The absolute As IVBA values were significantly higher in PM_10_ than in PM_150_ (paired Wilcoxon test, *p* = 2.5 × 10^−10^) and strongly correlated between the fractions (*R* ^*2*^ = 0.91, *p* < 0.001). In contrast, the relative IVBA did not differ significantly between the fractions (*p* = 0.309; Table S4 in Supporting Information S2), indicating comparable bioaccessible proportions despite higher total As concentrations in PM_10_. The differences in the absolute As IVBA therefore primarily reflect the elevated As concentrations in the finer fraction rather than differences in relative bioaccessibility. Significant regional differences were observed in the absolute As IVBA, and the relative IVBA also differed significantly among the regions, although the overall variability in % values was smaller (Table S5 in Supporting Information S2). Both the Kruskal–Wallis test and subsequent Dunn post‐hoc comparisons indicated that the relative As IVBA was significantly elevated only in Kutná Hora, while all the other regions were statistically comparable (Table S5 in Supporting Information S2).

The P extracted under gastric conditions (P_gast_) ranged from 39 to 2837 mg kg^−1^ in PM_150_ and from 56 to 5309 mg kg^−1^ in PM_10_, corresponding to 4.9%–89% and 6.5%–89% of the total P in PM_150_ and PM_10_, respectively (Figure S8a; Table S3 in Supporting Information S2). The relative extractability of P was approximately three times higher than the relative As IVBA. In contrast, the Fe extractability under gastric conditions (Fe_gast_) was low (<5.7% of total Fe in both fractions), suggesting the limited dissolution of Fe (oxyhydr)oxides during the IVBA assay (Figure S8b in Supporting Information S1). Small, but statistically significant, differences in the absolute and relative P_gast_ were observed between the regions (Table S6 in Supporting Information S2), while regional differences in the Fe_gast_ were minor (Table S7 in Supporting Information S2).

## 4 Discussion

### 4.1 Distribution and Mineralogy of Arsenic in Agricultural Soils

The agricultural soils investigated in this study exhibited highly variable As concentrations (25.5–2007 mg kg^−1^), reflecting both geogenic enrichment and contamination associated with historical mining and smelting. Most samples exceeded the Czech agricultural soil threshold of 40 mg kg^−1^, indicating widespread enrichment across the studied regions. Arsenic was preferentially concentrated in the exposure‐relevant PM_150_ and PM_10_ fractions, indicating potential for human exposure through soil ingestion and fugitive dust generation (Smith & Lee, 2003; US EPA, 2017). The variability in As enrichment among particle‐size fractions was partly related to the distribution of total Fe (*R* ^*2*^ = 0.39, *p* < 0.001), which likely reflects the abundance of reactive Fe (oxyhydr)oxides controlling As retention in oxic soils (Cornell & Schwertmann, 2003). In several well‐developed soils (Table S3 in Supporting Information S2), the incorporation of Fe (oxyhydr)oxides into large stable aggregates may have limited their accumulation in the finest fractions and consequently reduced the enrichment of As in exposure‐relevant particles (Cornell & Schwertmann, 2003; Schaetzl & Thompson, 2015).

In all the studied soils, Fe (oxyhydr)oxides dominated the As‐bearing phases. The EPMA indicated that the highest As concentrations occurred in poorly crystalline Fe (oxyhydr)oxides such as ferrihydrite, consistent with previous spectroscopic studies from several of the studied regions (Drahota et al., 2009, 2018, 2023; Filippi et al., 2007). However, the oxalate extraction revealed that, in most soil samples (68%; *n* = 171), the poorly crystalline Fe (oxyhydr)oxides accounted for less than 50% of the total As. This indicates that a substantial proportion of As is associated with more crystalline Fe (oxyhydr)oxides (goethite and hematite) or other relatively stable minerals (Drahota et al., 2014), where As may occur in less reactive coprecipitated or structurally incorporated forms rather than as surface‐bound species. Notable regional differences were observed, with poorly crystalline Fe (oxyhydr)oxides hosting the majority of As in up to 90% of the samples from Kutná Hora, suggesting a larger proportion of potentially reactive As forms in this region. Such partitioning between poorly crystalline and more crystalline Fe phases likely influences the reactivity and potential mobilization of As under competitive sorption conditions. Discrete As‐bearing minerals occurred only locally and represented a substantially smaller proportion of the total As relative to Fe (oxyhydr)oxides. These phases were mainly associated with historical mining and smelting contamination or localized weathering of primary As sulfides. Their limited abundance indicates that bulk As mobility and bioaccessibility in the studied agricultural soils are controlled predominantly by surface‐bound As associated with Fe (oxyhydr)oxides rather than by discrete (hydroxy)arsenate and hydroxysulfate minerals.

### 4.2 Controls on Potential Arsenic Mobility

**Figure 4:**
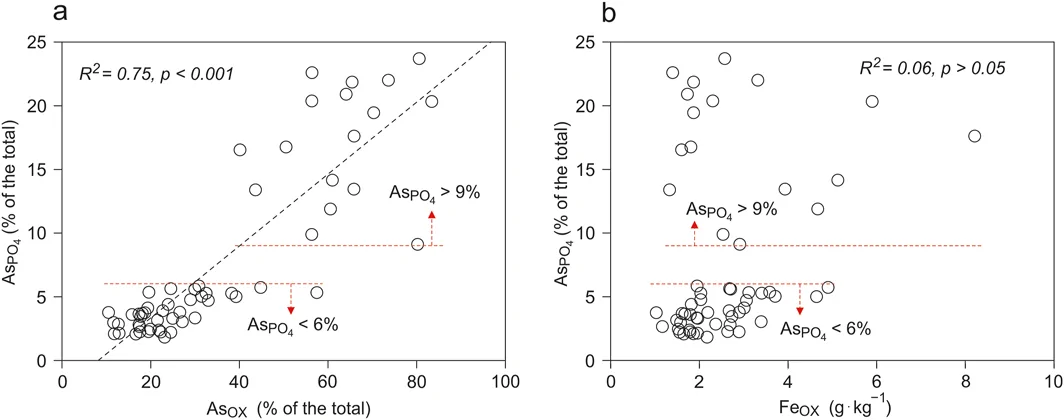
Relationship between the fraction of the surface‐bound As (As_PO4_) and (a) the fraction of As (As_OX_) associated with poorly crystalline Fe (oxyhydr)oxides, and (b) the content of Fe (oxyhydr)oxides (Fe_OX_) in 57 agricultural soils. The linear regression indicates a strong correlation in panel (a), whereas no significant relationship is observed in panel (b). Horizontal red lines with arrows separate the samples with As_PO4_ fractions lower than 6% and higher than 9%.

The applied phosphate and sulfate extractions represent operationally defined As fractions, interpreted as surface‐bound and potentially mobile As pools, respectively (Keon et al., 2001; Wenzel et al., 2001, 2002). These extractions indicated that the proportions of surface‐bound and potentially mobile As varied considerably between and within the regions (Figure S3a in Supporting Information S1), reflecting the substantial heterogeneity in As partitioning at the field scale. The surface‐bound As fraction was closely related to the oxalate‐extractable As, but did not show a clear correlation with the amount of oxalate‐extractable Fe (Figure 4). This indicates that the variability in the surface‐bound As cannot be attributed solely to the abundance of poorly crystalline Fe (oxyhydr)oxides, but also reflects differences in the contamination sources and sorption history.

**Figure 5:**
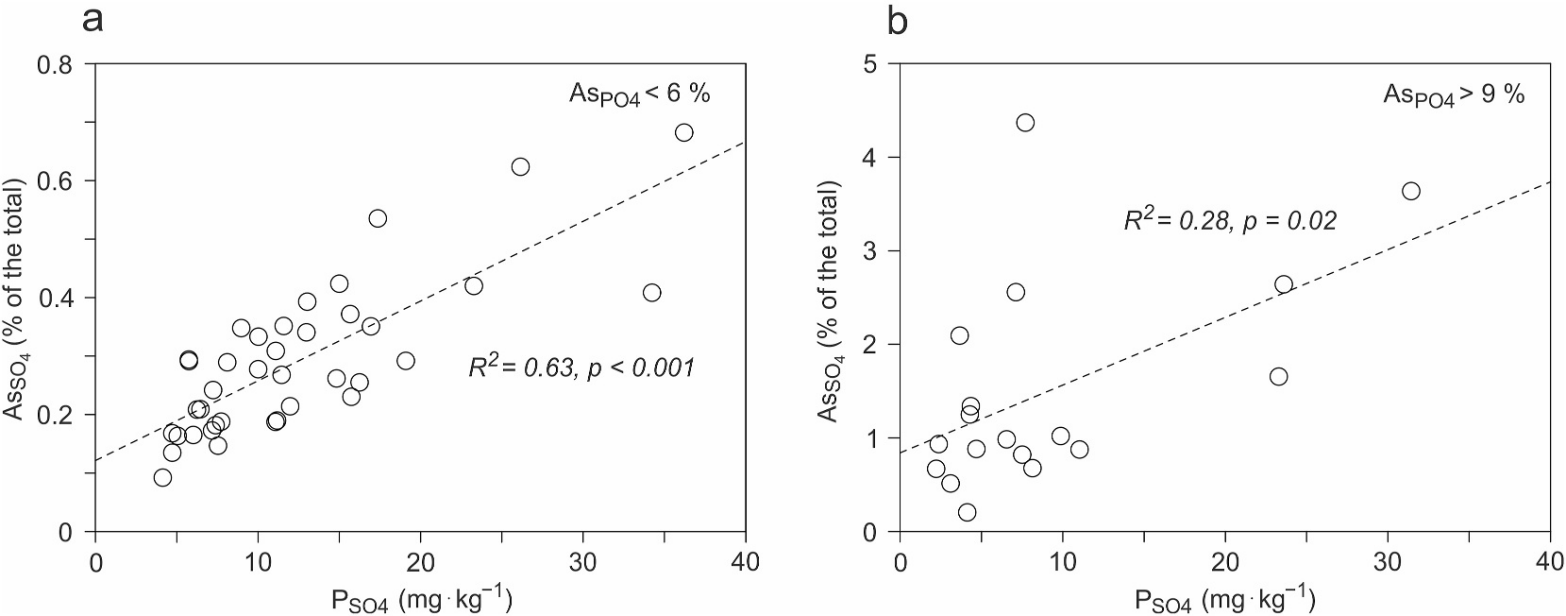
Relationship between the sulfate‐extractable As (As_SO4_) and P (P_SO4_) in the soils with (a) a low fraction of surface‐bound As (As_PO4_ < 6%) and (b) a high fraction of surface‐bound As (As_PO4_ > 9%). The linear regression shows strong correlation for soils with low surface‐bound As (a) and weak correlation for soils with high surface‐bound As (b), primarily driven by the outlying points.

The abundance of surface‐bound As strongly influenced the potential mobility of As in agricultural soils. Soils with a low surface‐bound fraction (As_PO4_ < 6%), representing approximately 70% of the samples, generally exhibited low As mobility (As_SO4_ < 0.68%) and strong coupling between the sulfate‐extractable As and P (Figure 5a), indicating that competitive sorption with phosphate was the dominant mechanism controlling the As mobilization. In contrast, the soils enriched in surface‐bound As (As_PO4_ > 9%) displayed substantially higher As mobility (*x̅* ± *σ*: As_SO4_ = 1.5 ± 1.1%) that was less dependent on dissolved phosphate concentrations (Figure 5b). The elevated As mobility was therefore not controlled solely by competitive sorption with phosphate. This behavior was particularly evident in soils from Kutná Hora, where elevated proportions of surface‐bound As coincided with enhanced As mobility (Figure S3a in Supporting Information S1). Although the relatively high soil pH in this region may have contributed to weaker As adsorption to Fe (oxyhydr)oxides and most clay minerals (Dixit & Hering, 2003; Goldberg, 2002; Jain & Loeppert, 2000), the large surface‐bound As pool likely primarily reflects historical smelting activities, including dispersed As‐bearing slags and smelter‐derived emissions, that supplied substantial amounts of As subsequently retained on mineral surfaces (Horák & Hejcman, 2016). Similar behavior observed in several acidic soils affected by historical smelting inputs or by redox‐driven cycling of Fe (oxyhydr)oxides associated with groundwater fluctuations (Drahota et al., 2021; Mansfeldt & Overesch, 2013) further indicates that contamination history and the abundance of surface‐bound As exert stronger controls on As mobility than pH alone.

Overall, these results demonstrate that the potential mobility of As in agricultural soils is primarily controlled by the abundance of surface‐bound As, which reflects the contamination history and pedogeochemical processes. Competitive sorption with phosphate represents an important short‐term mobilization mechanism, whereas pH likely modulates sorption equilibria and affects the mobility of As associated with mineral surfaces. These findings further indicate that long‐term phosphate inputs in agricultural soils may preferentially enhance As mobilization in soils enriched in surface‐bound As pools, consistent with previous studies reporting phosphate‐induced As release in contaminated agricultural systems (Cao & Ma, 2004; Davenport & Peryea, 1991; Peryea & Kammereck, 1991; Zafeiriou et al., 2019).

### 4.3 Soil Controls on Arsenic Bioaccessibility

Arsenic mineralogy is generally considered an important control of As bioaccessibility in soils (Basta & Juhasz, 2014; Sowers et al., 2022). In the studied agricultural soils, As is predominantly associated with Fe (oxyhydr)oxides of variable crystallinity, while discrete (hydroxy)arsenate and hydroxysulfate minerals were only locally present and accounted for a minor fraction of the total As. Although these As‐rich phases differ markedly in their solubility under gastric conditions (e.g., As IVBA of ∼70% for yukonite compared to ∼0.3% for pharmacosiderite; Drahota et al., 2025), their overall contribution to the total soil As was too limited to influence the bulk As IVBA significantly.

The controls on As bioaccessibility were evaluated using complementary correlation and regression analyses integrating the pH, total element concentrations and operationally defined As, Fe and P fractions. Both statistical approaches showed that As IVBA was controlled mainly by operationally defined As fractions (sulfate‐, phosphate‐, and oxalate‐extractable As) rather than by total soil As concentrations, consistent with previous studies (Cornejo‐Ponce & Acarapi‐Cartes, 2011; Juhasz et al., 2007; Meunier et al., 2011; Mikutta et al., 2014; Palumbo‐Roe et al., 2015; Smith et al., 2008). In particular, Spearman's analysis revealed the strongest positive correlations between the absolute As IVBA and phosphate‐extractable As (Table S8 in Supporting Information S2), with the regression models identifying As_PO4_ as the principal predictor of both the absolute and relative As IVBA (Tables S9 and S10 in Supporting Information S2). The correlation coefficients reached *ρ* ≈ 0.91–0.95, and the multiple regression models explained more than 90% of the variability in the absolute As IVBA. These results indicate that As IVBA was controlled predominantly by surface‐bound As likely associated with reactive Fe (oxyhydr)oxide surfaces. This interpretation is consistent with previous observations that geogenic As, commonly incorporated or coprecipitated within stable Fe (oxyhydr)oxides, is characterized by lower As IVBA, whereas higher As IVBA is typical of anthropogenically contaminated soils enriched in more reactive As associated with mineral surfaces (Juhasz et al., 2007; Palumbo‐Roe & Klinck, 2007; Smith et al., 2009).

In addition to the extractable As fractions, phosphorus showed the strongest secondary relationships with As IVBA. Positive correlations between As IVBA and P_gast_ (*ρ* ≈ 0.47–0.50, *p* < 0.001; Table S8 in Supporting Information S2) indicate the simultaneous mobilization of As and P under gastric conditions. The EPMA analyses indicated no systematic co‐occurrence of As and P in the soil minerals, suggesting that the observed relationship does not reflect dissolution of common As‐ and P‐bearing minerals, but rather competitive As and P interactions during the IVBA assay. Similar increases in As bioaccessibility induced by phosphate have been reported in laboratory amendment studies (Basta et al., 2007; Li et al., 2017), supporting the interpretation that phosphate competition for sorption sites promotes As release from reactive Fe (oxyhydr)oxide surfaces during IVBA assays, analogous to the mechanism identified in the sulfate extractions. However, unlike the short‐term phosphate amendment experiments, the present study demonstrates that long‐term agricultural phosphate accumulation can exert contrasting effects on As bioaccessibility depending on phosphate availability and surface association. In particular, higher P_OLSEN_ remained a significant negative predictor of As IVBA after accounting for the dominant effect of As_PO4_ and other variables in the regression models (Tables S9 and S10 in Supporting Information S2). This relationship suggests that long‐term phosphate accumulation may reduce As bioaccessibility through the occupation of reactive sorption sites and/or stabilization of poorly crystalline Fe (oxyhydr)oxides (Hongshao & Stanforth, 2001; Majzlan, 2011; Manning & Goldberg, 1996; Tiberg et al., 2020).

The Fe and pH also showed significant relationships with the As IVBA. Spearman's analysis revealed a weak negative correlation between the relative As IVBA and total Fe (Table S8 in Supporting Information S2), while regression models identified oxalate‐extractable Fe as secondary predictors of As IVBA (Tables S9 and S10 in Supporting Information S2). This pattern is consistent with previous studies showing that poorly crystalline Fe (oxyhydr)oxides can suppress As bioaccessibility through sorption processes (Mikutta et al., 2014; Palumbo‐Roe et al., 2015; Rodriguez et al., 2003). The Fe released during the gastric test represented only a small fraction of the total Fe in the soil, indicating limited dissolution of Fe (oxyhydr)oxides during the IVBA assay. Combined with the strong relationship between As IVBA and surface‐bound As fractions, these results suggest that As release was controlled primarily by desorption from reactive Fe (oxyhydr)oxide surfaces rather than by extensive dissolution of Fe host phases. The positive relationships between the relative As IVBA and pH (*ρ* up to ∼0.7; Tables S8–S10 in Supporting Information S2) further indicate that increasing pH weakens As adsorption to Fe (oxyhydr)oxide surfaces and thereby enhances As release (Juhasz et al., 2007; Smith et al., 2008; Yang et al., 2002).

### 4.4 Influence of Phosphate on Arsenic Mobility and Bioaccessibility

The studied agricultural soils exhibited elevated P_OLSEN_ concentrations (21–144 mg kg^−1^; median 54 mg kg^−1^) that exceeded the recommended agronomic target ranges for European agricultural soils (Jordan‐Meille et al., 2012), indicating substantial long‐term phosphate enrichment likely associated with repeated fertilization. Under these conditions, different pools of reactive phosphate appear to exert contrasting controls on As mobility and bioaccessibility through competitive interactions on Fe (oxyhydr)oxide surfaces. The readily soluble P was consistently associated with enhanced As release in both soil and gastric extractions, supporting competitive desorption as an important short‐term mobilization mechanism. The P concentrations measured in the sulfate (5–50 μmol L^−1^) and gastric extracts (13–1,720 μmol L^−1^) further support this interpretation because they fall within the range previously shown to promote ligand exchange on Fe (oxyhydr)oxide surfaces (Drahota et al., 2023; Goh & Lim, 2005; Mihaljevič et al., 2009; Venhauerova et al., 2022). In contrast, higher Olsen‐extractable P was associated with lower As IVBA in the regression models (Tables S9 and S10 in Supporting Information S2). This inverse relationship suggests that sustained phosphate accumulation promotes the progressive occupation of reactive sorption sites on Fe (oxyhydr)oxide surfaces (Hongshao & Stanforth, 2001; Manning & Goldberg, 1996; Tiberg et al., 2020), thereby decreasing the susceptibility of surface‐bound As to release under the gastric conditions. In addition, phosphate adsorption may thermodynamically stabilize Fe (oxyhydr)oxides and reduce their solubility relative to As‐enriched or anion‐free phases (Majzlan, 2011), which may further limit As release.

In agricultural soils receiving repeated fertilizer inputs, long‐term phosphate accumulation and interactions with soil sorbents are likely to influence the proportion of As mobilized under gastric conditions. Therefore, phosphate availability should be considered an important geochemical parameter in the assessment of As mobility and human exposure in agricultural soils.

### 4.5 Exposure Implications of Particle‐Size Dependent Arsenic Bioaccessibility

**Figure 6:**
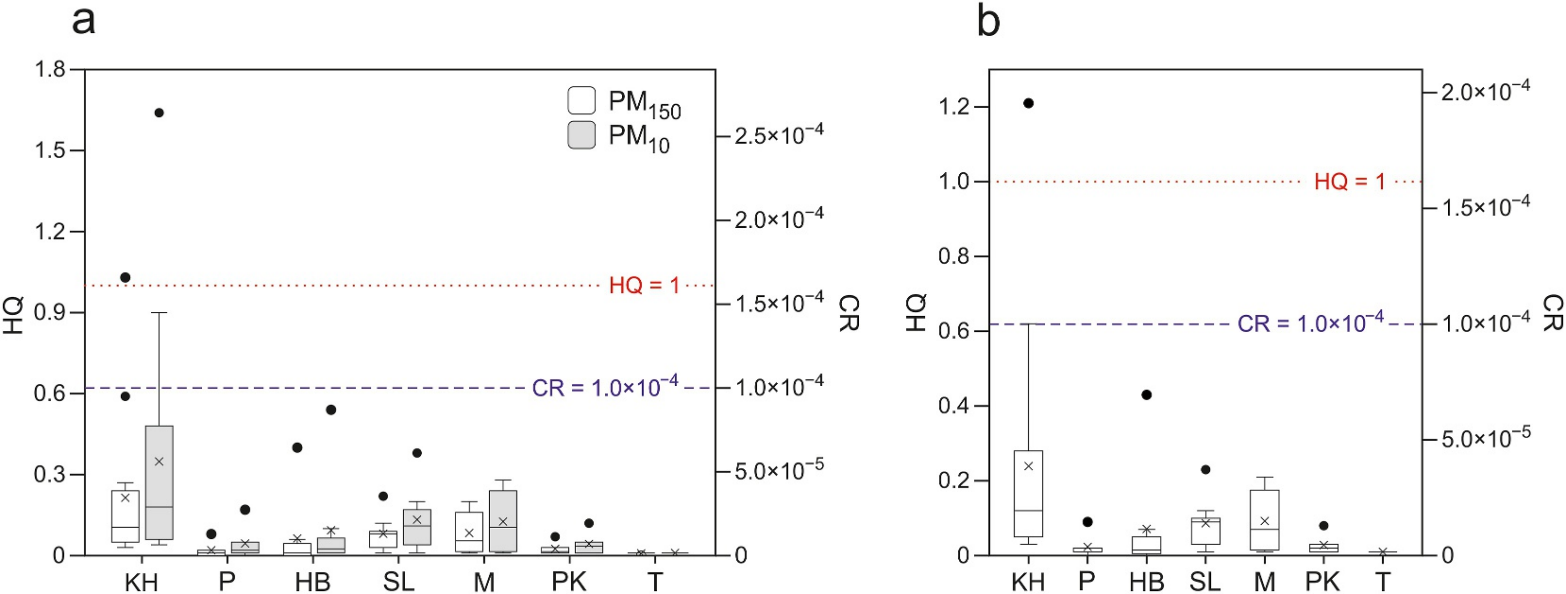
Box‐and‐whisker plots of the non‐carcinogenic (hazard quotient (HQ)) and carcinogenic risk (CR) indices derived from the chronic daily intake for particle‐size fractions PM_150_ and PM_10_ (a) and HQ and CR adjusted by the mass fraction of PM_10_ within PM_150_ (b). The study regions include Kutná Hora (KH), Příbram (P), Havlíčkův Brod (HB), Smolotely‐Líšnice (SL), Mokrsko (M), Příčovy (PK) and Tehov (T). The horizontal dotted line indicates HQ = 1, and the dashed line represents CR = 10^−4^, separating the samples with elevated non‐carcinogenic or carcinogenic risk, respectively. The boxes represent the interquartile range with the median (horizontal line) and mean (cross); the whiskers indicate values within 1.5× IQR; points denote outliers.

Figure 6 summarizes the CR and HQ values for PM_150_ and PM_10_ across the studied regions; the corresponding CDI values are listed in Table S3 in Supporting Information S2. In general, most agricultural soils did not exceed the non‐carcinogenic risk threshold (HQ = 1) when the bioaccessible As concentrations were considered, indicating that despite the elevated total As concentrations, the effective oral exposure is substantially moderated by the limited bioaccessibility. However, localized hotspots with elevated exposure potential were identified. The highest HQ and CR values were observed in the Kutná Hora region, particularly in sample KH4 collected from an agricultural field immediately adjacent to residential buildings in Kutná Hora (Figure S1 in Supporting Information S1). This sample exceeded the non‐carcinogenic threshold in both particle size fractions, and also exceeded the carcinogenic risk threshold, while an additional sample (KH3) exceeded the carcinogenic threshold only in PM_10_ (Table S3 in Supporting Information S2). These findings indicate that even within predominantly agricultural landscapes, localized areas with an elevated exposure potential may occur, particularly where historical mining and smelting activities have contributed to the accumulation of reactive surface‐bound As.

The particle size represents an important factor in the exposure assessment because it influences the dominant exposure pathway and the distribution of As among exposure‐relevant soil fractions. The PM_150_ fraction contributes primarily to incidental ingestion through hand‐to‐mouth contact, whereas PM_10_ can become airborne as fugitive dust during wind erosion or agricultural tilling. The CR and HQ values were generally higher in the PM_10_ fraction than in the PM_150_ fraction (Figure 6a and Table S3 in Supporting Information S2), primarily reflecting the elevated As concentrations in the finer fraction. For the integrated risk estimation, PM_10_ and the remaining PM_150_ fraction were combined according to the relative proportion of PM_10_ within PM_150_. The recalculated values indicate that the agricultural soils from Kutná Hora, Smolotely‐Líšnice, and Mokrsko exhibit systematically higher median HQ and CR values than soils from the other regions (Figure 6b), although the exceedance of the regulatory risk thresholds remained limited to a single hotspot sample KH4 from Kutná Hora.

It should be noted that the applied exposure parameters represent a conservative rural scenario consistent with regulatory practice. Although a default adult soil ingestion rate of 100 mg day^−1^ was applied according to the established regulatory guidance, quantitative soil ingestion data for agricultural populations remain limited and are commonly extrapolated from urban or suburban studies (Doyle et al., 2010). Recent mass‐balance studies conducted in rural and agricultural populations have reported a mean adult soil ingestion rate of approximately 30–75 mg day^−1^, with the 90th percentile values between 150 and 210 mg day^−1^ (Doyle et al., 2012; Irvine et al., 2014). The exposure scenario applied in this study therefore represents a precautionary, but still realistic, upper‐range exposure scenario for agricultural environments affected by elevated As concentrations. Children may experience higher exposure because of increased soil ingestion rates and hand‐to‐mouth behavior; therefore, the present assessment likely underestimates exposure in sensitive populations.

## 5 Conclusions

This study provides a field‐based evaluation of As mobility and bioaccessibility in agricultural topsoils affected by geogenic As enrichment, historical mining activities, and long‐term phosphate fertilization. Across the studied regions, As was predominantly associated with Fe (oxyhydr)oxides, whereas discrete As‐bearing minerals occurred only locally and represented minor proportions of the total soil As. Although the total As concentrations frequently exceeded national soil thresholds, most soils exhibited relatively low to moderate As bioaccessibility (2%–38%), consistent with the predominance of As associated with Fe (oxyhydr)oxides. The combined geochemical, mineralogical, and statistical results indicate that As mobility and bioaccessibility are controlled predominantly by reactive surface‐bound As fractions rather than by total soil As concentrations alone. Surface‐bound As was particularly elevated in the soils influenced by historical smelting activities, indicating that the contamination history and sorption processes strongly influence the abundance of reactive As pools and the resulting exposure potential. Phosphate exerted contrasting effects on As mobility and bioaccessibility depending on phosphate availability and surface association. Readily soluble phosphate fractions promoted As release through competitive desorption, whereas higher Olsen‐extractable P was associated with lower As bioaccessibility, indicating that long‐term phosphate accumulation may partially stabilize surface‐bound As. These findings indicate that phosphate should not be treated as a single geochemical variable in exposure assessment because distinct phosphate pools exert fundamentally different controls on As mobility and bioaccessibility. Despite elevated total As concentrations (up to 2000 mg kg^−1^), the risk assessment based on bioaccessible As indicated that most soils did not exceed non‐carcinogenic thresholds under the applied conservative rural exposure scenario. The study therefore highlights the importance of integrating particle‐size distribution, geochemical partitioning, phosphate availability, and bioaccessibility measurements into the assessment of human exposure in agricultural soils affected by natural and anthropogenic As contamination.

## Conflict of Interest

The authors declare no conflicts of interest relevant to this study.

## Supporting information

Supporting Information S1

Supporting Information S2

## Data Availability

Supporting data for this study, including soil geochemical characterization, selective extraction results, arsenic bioaccessibility measurements, mineralogical data, and exposure assessment outputs, are available in (Drahota, 2026). Statistical analyses were performed using R version 4.5.1 (R Core Team, 2025) within the RStudio environment. Data visualization was prepared using DataGraph 5 (Visual Data Tools, USA).
