# Supplementary material for: Contrasting Effects of Phosphate on Arsenic Mobility and Human Exposure in Agricultural Soils
Source: Geohealth. 2026 Sep 15;10(9):e2026GH002031. doi: 10.1029/2026GH002031 (PMC13575764; doi:10.1029/2026GH002031)
Supplement: Supplementary file 1 [file GH2-10-e2026GH002031-s002.docx]

**Supporting Figures to:**

**ʻ****Contrasting effects of phosphate on arsenic mobility and human exposure in agricultural soilsʼ**

Petr Drahota^1^*, Alena Černíková^2,3^, Vojtěch Ettler^1^, Johana Kešnerová^1^, Martin Lichovník^1^, Martin Mihaljevič^1^

^1^Institute of Geochemistry, Mineralogy and Mineral Resources, Faculty of Science, Charles University, Albertov 6, 128 00 Prague 2, Czech Republic

^2^Institute of Applied Mathematics and Information Technologies, Faculty of Science, Charles University, Albertov 6, 128 00 Prague 2, Czech Republic

^3^Department of Mathematics, Faculty of Science, Jan Evangelista Purkyně University in Ústí nad Labem, Pasteurova 3632/15, 400 96 Ústí nad Labem

(10 Pages, 8 Figures)

**Table of contents**

1. Site locations	S2

2. Texture and pH	S3

3. Chemical extractions	S3

4. Total P	S4

5. Oxalate extractions	S5

6. Oxalate Fe/As and Fe/P	S6

7. EPMA analyses	S7

8. Gastric extractability of Fe and P	S8

*corresponding author: [petr.drahota@natur.cuni.cz](mailto:petr.drahota@natur.cuni.cz)

1. **Site locations**

**Figure S1**. Map of sampling sites in the Czech Republic. Aerial view of the sites (based on Google Earth^TM^) with the locations of the sampling points indicated by white dots.

2. **Texture and pH**

**Figure S2**. Box-and-whisker plots showing (a) the proportions of PM_150_ (white) and PM_10_ (gray) soil particle fractions, and (b) soil pH measured in deionized water (white) and in 1 M KCl solution (gray) from the studied areas (KH – Kutná Hora, P – Příbram, HB – Havlíčkův Brod, SL – Smolotely-Líšnice, M – Mokrsko, PK – Příčovy-Krásná Hora, T – Tehov). The box plots show the median (horizontal line), interquartile range (IQR; box), minimum and maximum values within 1.5× IQR (whiskers), mean (cross), and outliers (individual points).

3. **Chemical extractions**

**Figure S3**. Box-and-whisker plots showing the chemical fractionation of (a) As extracted using sulfate (SO₄) and phosphate (PO₄) and (b) P extracted using sulfate (SO₄) and Olsen (OLSEN) methods from 57 soil samples. The study sites include Kutná Hora (KH), Příbram (P), Havlíčkův Brod (HB), Smolotely-Líšnice (SL), Mokrsko (M), Příčovy (PK), and Tehov (T).

4. **Total P**

**Figure S4**. Box-and-whisker plot showing the variability of P concentrations in 57 soil samples in three different particle size fractions (<2 mm, PM_150_ and PM_10_). The study sites include Kutná Hora (KH), Příbram (P), Havlíčkův Brod (HB), Smolotely-Líšnice (SL), Mokrsko (M), Příčovy (PK), and Tehov (T).

5. **Oxalate extractions**

**Figure S5**. Box-and-whisker plot showing the fractionation of As, P, and Fe in oxalate extractions of 57 soil samples in three different particle-size fractions (<2 mm, PM_150_ and PM_10_). The study sites include Kutná Hora (KH), Příbram (P), Havlíčkův Brod (HB), Smolotely-Líšnice (SL), Mokrsko (M), Příčovy (PK), and Tehov (T).

6. **Oxalate Fe/As and Fe/P**

**Figure S6**. Box-and-whisker plot showing the molar Fe/As and Fe/P ratios from oxalate extractions of 57 soil samples in three different particle-size fractions (<2 mm, PM_150_ and PM_10_). The study sites include Kutná Hora (KH), Příbram (P), Havlíčkův Brod (HB), Smolotely-Líšnice (SL), Mokrsko (M), Příčovy (PK), and Tehov (T).

7. **EPMA analyses**

**Figure S7**. As_2_O_5_ vs Fe_2_O_3_ plot of secondary As-bearing mineral phases in selected PM_150_ samples. Identification of mineral groups is based on microprobe analyses. Mineral abbreviations: Bdn - beudantite; Bpsd - bariopharmacosiderite; Cmt - carminite; FHO - Fe(III) (oxyhydr)oxides; MHO - Mn (oxyhydr)oxides; Pjrs - plumbojarosite.

8. **Gastric extractability of Fe and P**

**Figure S8**. Box-and-whisker plots showing the absolute (left panel) and relative (right panel) gastric extractability of P (a) and Fe (b) across size fractions (PM_150_ and PM_10_) of 57 soil samples from Kutná Hora (KH), Příbram (P), Havlíčkův Brod (HB), Smolotely-Líšnice (SL), Mokrsko (M), Příčovy (PK), and Tehov (T).
